# Robust reference intervals for Liver function test (LFT) analytes in newborns and infants

**DOI:** 10.1186/1756-0500-5-493

**Published:** 2012-09-07

**Authors:** Mulugeta Melkie, Mahilet Yigeremu, Paulos Nigussie, Shawel Asrat, Tatek Gebreegziabher, Tilahun Teka, Samuel Kinde

**Affiliations:** 1 Department of Medical Laboratory Science, Arbaminch University, Arbaminch, Ethiopia; 2 Faculty of Medicine, Addis Ababa University, Addis ababa, Ethiopia; 3 Ethiopian Health and Nutrition Research Institute, Addis Ababa, Addis ababa, Ethiopia; 4 Tikur Anbessa Specialized Hospital, Addis Ababa, Addis ababa, Ethiopia

**Keywords:** Reference intervals, Liver function test, Robust method, Newborns, Infants, Cord blood

## Abstract

**Background:**

Reference intervals (RIs) are ranges of upper and lower limits of a given analyte which are used for a laboratory test to determine whether a disease is present or absent or to know if the patient is at risk for future disease states. In Ethiopia, a country with highly diversified population groups and geographical sites, there are no established RIs to metabolic analytes including the liver function test (LFT) analytes for the pediatric population though it has been known that liver function assessment in this population is vital as a result of varied vulnerability to both endogenous and xenobiotic substances.

**Methods:**

A cross sectional study was conducted in Tikur Anbessa Specialized Hospital (TASH) and Teklehaymanot Health Center (THC) from November 2010 to April 2011. 117 cord blood (from newborns) and venous blood samples (from infants) were collected and analyzed using HumaStar 300. All pre-analytical, analytical and post-analytical aspects were thoroughly controlled. A robust, CLSI/ IFCC recommended, method was used for the determination of upper and lower end points covering 95% of the reference values of each analyte with respective 90% CIs using MedCalc® software.

**Results:**

Combined RIs for newborns and infants were established for albumin, AST, ALP, direct bilirubin and total bilirubin to be 3.88-5.82 g/dl, 16.1-55.4U/l, 130-831U/l, <0.41 mg/dl and <1.37 mg/dl respectively. But, separated RIs were indicated for ALT and GGT as 1.2-23.1U/l and 6.94-24.8U/l ALT; and 30.6-160.7U/L and 10–28.2U/l GGT for newborns and infants respectively. Some maternal and infantile factors were identified to affect the values of analytes.

**Conclusion:**

Almost all analytes were different from previously reported values for other target population of similar age group, kit insert values and adult values. So, interpretation of values of these analytes in newborns and infants of Ethiopian population sounds better to be performed by using such RIs taking the effect of some maternal and infantile factors in to account.

## Background

It is well known that reference intervals (RIs) depend on many factors including the type of instrument and reagents used, the principle or method for the test that is being performed, the type of population being served and the strength of quality assurance practiced at the laboratories [1]. Hence, establishing RIs continued to be a major concern in many parts of the world since the RIs in-use were established mainly for the Caucasian population, and are often inappropriate for the diverse population that many laboratories serve, and existing RIs need to be verified or validated as they were established decades ago with methods and instrumentations that are now obsolete [2]. Moreover, establishment of RIs is becoming one agenda in assurance of quality with in clinical laboratories [3,4].

In Ethiopia, a country with highly diversified population groups and geographical sites, there are no established RIs for metabolic analytes including the liver function test (LFT) analytes for the pediatric population although some efforts have been initiated for the adult population [5]. It has been known that liver function assessment in pediatric population is vital since there is varied vulnerability to both endogenous and xenobiotic substances which is resulted from developmental changes in the liver’s metabolic activity. Prolonged action of drugs in newborns is attributed to the decreased capacity of the liver to metabolize, detoxify and excrete xenobiotics. Neonatal jaundice is also resulted because of decreased capacity of glucuronide conjugation [6]. As a result, clinicians and researchers have been adopting RIs from textbooks and package inserts that were originally established based on Caucasian population for the interpretation of pediatric laboratory results though it is known that using non-Ethiopian RIs may be misleading.

The purpose of this study is then to establish RIs for the commonly requested LFT analytes in Ethiopian newborns and infants (≤ 1 year) and to determine the effect of selected demographic variables on the values of these analytes.

## Method and materials

A cross sectional study was conducted in Tikur Anbessa Specialized Hospital (TASH), department of Obstetrics and Gynecology (OBGY) and Teklehaymanot Health Center (THC) from November 2010 to April 2011. Newborns delivered in OBGY department of TASH with over 37 weeks of gestation, ≥2500 g birth weight and no history of fetal problems were enrolled in the study and their cord blood was collected. Preterm newborns, newborns with <2500 g birth weight, newborns requiring intensive resuscitation and care, newborns from mothers with documented ante- and/or intra-partum complications (gestational diabetes, HIV, hepatitis B/C, eclampsia, etc.) were excluded. Similarly, infants coming for immunization to THC without any organic diseases or other diseases that can alter their biochemical profile and those with weights > 2700 g were included in the study and venous blood was collected. Infants that have organic diseases and other diseases that alter their biochemical profile were excluded from the study. Infants who have taken medications that can affect their biochemical profile were also excluded from the study. Structured questionnaires were used in both cases for the collection of selected demographic information.

The proposal of this study was reviewed by the Department of Medical Laboratory Sciences research review committee and by the Institutional Review Board (IRB) of Addis Ababa University, Faculty of Medicine. Informed (written) consent was obtained from mothers before specimen and data collection.

The pre-analytical, analytical and post-analytical phases of the analyses were controlled throughout the study. Moreover, a pilot study was conducted before the actual data collection. HumaStar 300 analyzer **(**Human diagnostics worldwide, Germany) was used for laboratory analyses and the methods applied for determination of analytes are summarized in Table 1.

**Table 1:** Summary of test methods applied for each analyte

**LFT analytes**	**Analytical method**
Aspartate aminotransferases (AST)	IFCC Modified method, 37^o^C [7]
Alanine aminotransferases (ALT)	IFCC Modified method, 37^o^C [8]
γ- glutamyltransferases (GGT)	γ-glutamyl-*p*-nitroanilide substrate [9]
Alkaline Phosphatase (ALP)	Optimized standard method [10]
Bilirubin (Direct + Total)	Modified Jendrasic/Grof method [11]
Albumin	BCG method [12]

*LFT* = Liver function tests, *IFCC* = International federation for Clinical Chemistry, *BCG* = Bromocresol green.

The data obtained from the analyzer and the questionnaires were entered in Microsoft excel sheet and analyzed by MedCalc® software Version 12.1.3. Tukey test and D’Agostino-Pearson (DAP) test were used to detect outlier values and to determine normality of distributions of analyte values respectively. The effects of different variables on the values of the analytes were determined through ANOVA and independent sample t-test. Then, the upper and lower end points covering 95% of the reference value of the analytes were determined with their respective 90% confidence intervals (CIs) by using the robust method, according to the IFCC/CLSI recommendation [13]. Haris and Boyd rule was applied to determine whether partitioning of RIs should be done.

## Result

### Analytical performance of the methods

The maximum intra-assay coefficients of variations detected (CVs) were 4.15% and 4.31% for normal and pathological control sera respectively. Similarly, the maximum inter-assay CVs were 3.31% and 4.95% for normal and pathological control sera respectively (Table 2). Commercial quality control sera (HUMATROL N and P) were included in every session of analyses. LJ charts were then plotted and all the quality control results were in the acceptable limits.

**Table 2:** Intra- and inter assay CVs determined from duplicate analysis of quality control sera (HUMATROL N and P) for LFT analytes

*** Analytes***	***Intra assay CVs***		***Inter assay CVs***	
	***Normal control***	***Pathological control***	***Normal control***	***Pathological control***
AST	2.42%	1.01%	**3.31%**	4.15%
ALT	0.63%	1.94%	3.11%	1.65%
GGT	1.50%	2.72%	2.86%	3.01%
ALP	1.83%	1.63%	1.58%	2.48%
Direct Bilirubin	2.00%	3.32%	2.93%	**4.95%**
Total Bilirubin	**4.15%**	**4.31%**	1.89%	3.64%
Albumin	0.89%	0.89%	3.07%	2.46%

*CVs* = Coefficient of variations, *LFT* = Liver function tests, *N* = normal, *P* = pathological, *AST* = Aspartate aminotransferases, *ALT* = Alanine aminotransferases, *GGT* = γ- glutamyltransferases, *ALP* = Alkaline Phosphatase.

### Demographic data of study participants

A total of 117 study participants that fulfill the inclusion criteria were included in this study among which 60 (51.3%) were newborns and 57 (48.7%) were infants. Overall, male study participants accounted for 53.8% where as females accounted for 46.2%. Summary of the other selected demographic variables is presented in Tables 3 and 4.

**Table 3:** summary of the distribution of selected demographic variables among newborns and infants

**Demographic variables**	**Newborns (n = 60)**	**Infants (n = 57)**	***p*-value**
**Height**	$\overset{–}{x}$ = 50.9 cm SD = 3.1 cm	$\overset{–}{x}$ = 60.5 cm SD = 8.5 cm	
**Weight**	$\overset{–}{x}$ = 3120.0 g SD = 411.7 g	$\overset{–}{x}$ = 6956.1 g SD = 1996.2 g	
**Head circumference**	$\overset{–}{x}$ = 35.3 cm SD = 3.1 cm	$\overset{–}{x}$ = 43.7 cm SD = 3.3 cm	
**Mode of deliveries**			
Normal vaginal delivery	n = 43 (71.7%)	NA	NA
Cesarean section	n = 17 (28.3%)		
**Sex**			
Male	n = 32 (46.7%)	n = 31 (54.4%)	*P = 0.9431*^*§*^
Female	n = 28 (53.3%)	n = 26 (45.6%)	
**Maternal educational level**			
Unable to write and read	n = 7 (11.7%)	n = 7 (12.3%)	
Below 8^th^ grade	n = 22 (36.7%)	n = 34 (59.6%)	*P = 0.0380*^*§*^
High school complete	n = 16 (26.7%)	n = 11 (19.3%)	
Certificate and above	n = 15 (25%)	n = 5 (8.8%)	
**Maternal occupation**			
House wives	n = 41(68.3%)	n = 18 (31.6%)	
Employed	n = 10 (16.7%)	n = 7 (12.3%)	*P < 0.0001*^*§*^
Private business	n = 7 (11.7%)	n = 9 (15.8%)	
Jobless	n = 2 (3.3%)	n = 23 (40.4%)	
**Maternal parity**			
Primiparous	n = 39 (65%)	n = 27 (47.4%)	*P = 0.0826*^*§*^
Multiparous	n = 21 (35%)	n = 30 (52.6%)	
**Maternal alcohol consumption**			
Yes	n = 11 (18.3%)	n = 2 (4.3%)	*P = 0.0605*^*§*^
No	n = 49 (81.7%)	n = 44 (95.7%)	
**Maternal chat chewing**			
Yes	n = 0 (0%)	n = 3 (6.5%)	*P = 0.1568*^*§*^
No	n = 60 (100%)	n = 43 (93.5%)	
**Maternal cigarette smoking**			
Yes	n = 0 (0%)	n = 0 (0%)	*P = 0.2067*^*§*^
No	n = 60 (100%)	n = 57 (100%)	

^§^ chi-square test, *NA* = not available, *n* = number, *cm* = centimeter, *g* = gram, *x–* = mean, *SD* = standard deviation.

**Table 4:** Summary of feeding practice in infants of different age groups

***Foods***					
***Age groups***	***Breast milk only***	***Formula milk only***	***Combination of breast and formula milk***	***Additional foods***	***Total***
***< 6 months***	22 (78.6%)	2 (7.1%)	4 (14.3%)	0 (0.0%)	28 (49.1%)
***> 6 months***	7 (24.1%)	9 (31.0%)	7 (24.1%)	6 (20.7%)	29 (50.9%)
*** Total***	29 (50.9%)	11 (19.3%)	11 (19.3%)	6 (10.5%)	57 (100%)

### RI calculations for the LFT analytes

According to Tukey test, no outlier values were detected in all the studied analytes; and according to the DAP test, all the data were normally distributed/ were found to be normally distributed after logarithmic transformation.

**Figure 1:**
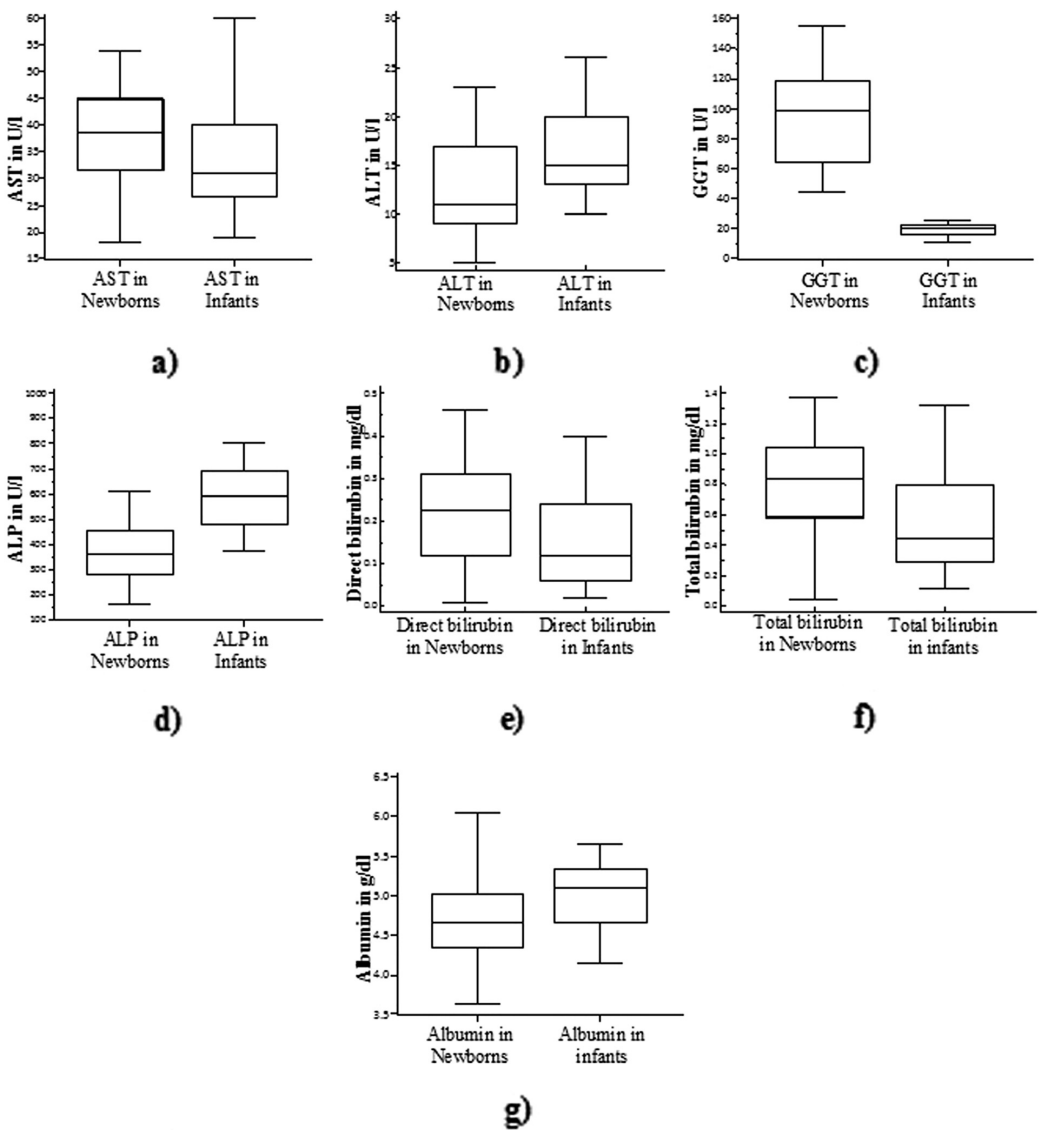
Box and Whisker plots indicating the difference among newborns and infants in values of AST (a), ALT (b), GGT (c) ALP (d), direct bilirubin (e), total bilirubin (f) and albumin (g).

All of the LFT analytes were different in newborns and infants though only ALT and GGT required separated RIs (Figure 1). The combined RIs (for newborns and infants) of AST, ALP, direct bilirubin, total bilirubin and albumin were 16.1-55.4 U/L, 130–831 U/L, below 0.41 mg/dl, below 1.37 mg/dl and 3.88-5.82 g/dl respectively. The separated RIs of ALT were calculated to be 1.2-23.1 U/L and 6.94-24.8 U/L for newborns and infants respectively; while the separated RIs of GGT were determined to be 30.6-160.7 U/L and 10–28.2 U/L respectively.

Maternal alcohol consumption during the periods of gestation was the only factor that affected the values of some LFT analytes (AST and ALP) in newborns (Table 5). But in infants, age group, gender and feeding practice influenced the values of some of LFT analytes (Table 6). Some of the effects were even strong enough to statistically dictate separated RIs.

**Table 5:** Results of independent sample t-tests(p-values) which were performed to see effect of maternal and neonatal factors on the values of LFT analytes in newborns

***Analyte***	***Variables***				
	***Sex***	***Maternal parity***	***Mode of delivery***	***Maternal alcohol consumption***	***Harris and Boyd***
AST	0.181	0.133	0.080	**0.003**	**Separate RI**
ALT	0.448	0.359	0.186	0.184	No separate RI
ALP	0.352	0.510	0.189	**0.011**	**Separate RI**
GGT	0.642	0.572	0.602	0.970	No separate RI
Direct bilirubin	0.846	0.933	0.452	0.455	No separate RI
Total bilirubin	0.935	0.745	0.561	0.180	No separate RI
Albumin	0.053	0.155	0.158	0.256	No separate RI

*LFT* = Liver function tests, *RI* = Reference interval, *AST* = Aspartate aminotransferases, *ALT* = Alanine aminotransferases, *GGT* = γ- glutamyltransferases, *ALP* = Alkaline Phosphatase.

**Table 6:** Results of independent sample t-tests* and ANOVA^**†**^**which were performed to see effect of maternal and infantile factors on the values of LFT analytes in infant blood samples**

*** Analyte***	***Variables***						
	***Age group***	***Sex***	***Feeding practice***	***Maternal parity***	***Maternal alcohol consumption***	***Maternal chat chewing***	***Harris and Boyd***
AST	0.514*	0.304*	0.397^†^	0.488*	0.241*	0.090*	No separate RI
ALT	0.080*	0.030*	0.354^†^	0.764*	0.522*	0.369*	**Separate RI**
ALP	0.002*	0.160*	**0.007**^†^	0.050*	0.678*	0.153*	No separate RI
GGT	0.000*	0.672*	0.928^†^	0.476*	0.810*	0.928*	**Separate RI**
Direct bilirubin	0.090*	0.572*	0.458^†^	0.673*	0.210*	0.622*	No separate RI
Total bilirubin	0.765*	0.585*	0.835^†^	0.143*	0.661*	0.714*	No separate RI
Albumin	0.001*	0.860*	0.168^†^	0.349*	0.111*	0.759*	**Separate RI**

*ANOVA* = analysis of variance, *LFT* = Liver function tests, *RI* = Reference interval, *AST* = Aspartate aminotransferases, *ALT* = Alanine aminotransferases, *GGT* = γ- glutamyltransferases, *ALP* = Alkaline Phosphatase.

Up on comparison of the RIs obtained from this study (Table 7) with previously reported values of similar age group, visually significant differences were seen. The values were also different from respective adult values determined for adult Ethiopian population (Table 8).

**Table 7:** Summary of the RIs LFT analytes in cord and infant blood samples

***Analyte***		***n***	***Min. value***	***Max. value***	***DAP test***	***Mean (95% CI)***	***Lower limit (90% CI)***	***Upper limit (90%CI)***	***RI***
**Albumin (g/dl)**	**Combined**	117	3.63	6.04	*P = 0.341*	4.84 (4.75-4.93)	3.88 (3.83-3.97)	5.82 (5.71-5.86)	**3.88-5.82**
	**Infants** < 6 months > 6 months	28 29	4.15 4.82	5.45 5.65	*P = 0.475 P = 0.463*	4.80 (4.66-4.95) 5.30 (5.22-5.39)	4.00 (3.85-4.12) 4.87 (4.72-4.95)	5.59 (5.50-5.76) 5.77 (5.71-5.89)	**4.00-5.59 4.87-5.77**
**AST (U/l)**	**Combined**	117	18	60	*P = 0.204*	35.9 (34.1-37.7)	16.1 (14.7-17.1)	55.4 (53.3-56.5)	**16.1-55.4**
	**Newborns** Alcohol Yes Alcohol No	11 49	34 18	54 53	*P = 0.43 P = 0.36*	45.5 (40.6-50.4) 36.2 (33.5-38.9)	28.2 (26.4-31.9) 17.4 (14.8-23)	63 (61.5-66.8) 55.7 (54.2-58.1)	**28.2-63 17.4-55.7**
**ALT (U/l)**	**Newborns**	60	5	23	*P = 0.101*	12.5 (11.1-13.8)	1.2 (0.08-3.06)	23.1 (21.4-24.8)	**1.2-23.1**
	**Infants** Male Female	57 31 26	10 10 10	20 23 26	*P = 0.095 P = 0.123 P = 0.435*	16.2 (15.1-17.3) 15.1 (13.8-16.4) 17.6 (15.7-19.4)	6.94 (4.92-9.04) 6.5 (5.0-7.7) 7.7 (7.1-9.1)	24.8 (22.9-26) 22.1 [14-17] 27.3 (25.6-29.7)	**6.94-24.8 6.5-22.17.7-27.3**
**ALP (U/l)**	**Combined**	117	163	954	*P = 0.141*	488 (456–520)	130 (112–155)	831 (781–848)	**130-831**
	**Newborns**								
	Alcohol Yes	11	163	418	*P = 0.89*	291 (241–341)	112 (78–133)	463 (435–513)	**112-463**
	Alcohol No	49	176	954	*P = 0.82*	385 (346–428)	180 (169–206)	808 (773–874)	**180-808**
**GG (U/l)**	**Newborns**	60	45	155	*P = 0.320*	96 (87.6-104)	30.6 (27.8-37.3)	160.7 (150–171)	**30.6-160.7**
	**Infants**	57	11	26	*P = 0.272*	19 (17.9-20.2)	10 (9.2-12.4)	28.2 (26.8-29.4)	**10-28.2**
	< 6 months	28	14	26	*P = 0.673*	21.2 (20–22.4)	14.8 (13.5-15.9)	27.9 (26.6-29.6)	**14.8-27.9**
	> 6 months	29	11	26	*P = 0.233*	17 (15.2-18.7)	6.9 (1.9-8.0)	26.1 (23.5-28.2)	**6.9-26.1**
**Direct bilirubin (mg/dl)**	**Combined**	117	0.01	0.46	*P = 0.075*	0.18 (0.16-0.20)	−0.06 [−0.07]-[−0.04]	0.41 (0.39-0.43)	**Below 0.41**
**Total bilirubin (mg/dl)**	**Combined**	117	0.04	1.37	*P = 0.130*	0.69 (0.62-0.75)	−0.01 [−0.01]-[0.02]	1.37 (1.31-1.40)	**Below 1.37**

*LFT* = Liver function tests, *RI* = Reference interval, *AST* = Aspartate aminotransferases, *ALT* = Alanine aminotransferases, *GGT* = γ- glutamyltransferases, *ALP* = Alkaline Phosphatase, *n* = number, *min* = minimum, *max* = maximum, *CI* = confidence interval, *DAP* = D’Agostino-Pearson.

**Table 8:** Comparing the RIs of LFTs in newborns and infants with values for the same age group and for adults in previous studies and kit inserts

	**AST (U/l)**	**ALT (U/l)**	**ALP (U/l)**	**GGT (U/l)**	**Direct bilirubin (mg/dl)**	**Total bilirubin (mg/dl)**	**Albumin (g/dl)**
**Current study**	16.1-55.4	Newborns = 1.2-23.1	130-831	Newborns = 30.6-160.7	Below 0.41	Below 1.37	3.88-5.82
		Infants = 6.94-24.8		Infants = 10–28.2			
**Perkins et al.**[18] (cord blood)	17-59	4-27	87-303	27-339			3.0-4.1
**Lockitch et al.**[19] (Up to 3 years)	20-60	5-45	145-320	6-19	Below 0.12	0.18-0.99	3.4-4.2
**Kit inserts**	Men ≤ 37 [20]	Men ≤ 42 [21]	Children ≤ 15 years ≤ 644 [22]	Men = 11-61 [23]	≤ 0.25 [14]	At birth ≤ 5 [14]	3.8-5.1 [15]
	Women ≤ 31 [20]	Women ≤ 32 [21]	Men = 80–306 [22]	Women = 9–39 [23]		5 days ≤ 12 [14]	
			Women = 64–306 [22]			1 month ≤ 1.5 [14]	
						Adults ≤ 1.1 [14]	
**Adult values**	14-60 [16]	8-61 [16]			0.02-0.52 [16]	0.17-2.16 [16]	5.8-8.8 [16]
	Men = 10-58 [5]	Men = 6–42 [5]	Men = 109–353 [5]	Men = 8.5-63 [5]	Men = 0.2-1.47 [5]	Men = 0.6-2.3 [5]	
	Women = 6-45 [5]	Women = 4-27.4 [5]	Women = 97–294 [5]	Women = 6–59 [5]	Women = 0.2-1.45 [5]	Women = 0.5-1.72 [5]	

*RI* = Reference interval, *LFT* = Liver function tests, *AST = Aspartate aminotransferases, ALT = Alanine aminotransferases, GGT = γ- glutamyltransferases, ALP = Alkaline Phosphatase.*

## Discussion

This study showed that the activity of AST in newborns was higher than in infants (*p = 0.026,* Figure 1a) even though separated RI was not justifiable according to Harris and Boyd rule. The increment could be due to the release of placental AST in to fetal circulation [17]. Both the upper and lower limits of this RI were similar to the previously reported values of the same age group population and to adult values. But, both upper and lower limits were higher than those provided in manufacturer kit inserts. Though the RI given on manufacture kit inserts lack traceability, it is clearly seen that the values are lower even than values given for adult let alone the pediatric population (Table 8).

According to Elinav et al., there is a significant association between age and serum ALT activity in which a characteristic inverted ‘U’ curve pattern is attained by ALT with age i.e. it increases up to 40–55 years and then decreases [24]. Accordingly, slightly lowered activity of ALT was found in newborns (Figure 1b) that necessitated separated RIs. This can be explained in the view that the fetus in normal physiological circumstances is dependent on the mother for frequent supply of glucose without a significant need of glucose production by gluconeogenesis [25]. But during infancy, there is increased gluconeogenesis and as a result the key enzyme in this process which converts alanine in to alpha ketoglutarate at the initial step, ALT, is increased [26]. The RIs determined for ALT were generally lower than values provided for children up to 3 years of age and adults (Table 8) which could be due to age difference, while they were comparable with cord blood values given in literature.

It has been documented that serum ALP activity can increase up to six times the upper reference limit of adult values during infancy and childhood, which then decreases to attain adult values by the age of 16–20 years the predominating isoenzyme being of bone origin [27]. Significant increment of ALP activity is detected in infants over newborns (*p = 0.0001,* Figure 1d) without a need for separated RIs. This RI was different from published values for similar age group population and adult population; particularly, the upper limit of the RI was higher (Table 8). As explained by Lucas et al., increased ALP activity might be related to slower growth rate [28].

Despite the fact that the increased activity is neither having any known clinical significance nor resulted from any fetal forms of the enzyme besides indicating immaturity [29], the activity of GGT was higher in newborns (*p < 0.0001*, Figure 1c) than in infants. In newborns, GGT activity may reach up to 5–7 times the upper limit of normal for adults [30]. The difference also required separated RIs. The RI of newborns was narrower than the value provided by Perkins et al. [18] in which the upper limit was almost a double of the current study while the lower limit was nearly equivalent. But, RI for infantile GGT activity was higher than the value given for children up to 3 years of age (Table 8) while the RI, more specifically, the upper limit was significantly lower than adult values as adults are known to have raised serum GGT activity induced from many factors including alcohol consumption [31].

As shown in Figure 1e and 1f, both direct and total bilirubin values were higher in cord blood than in infants (*p = 0.0067* and *p < 0.0001* respectively) although the differences failed to illustrate separated RIs. The increment in cord blood bilirubin could be resulted since only infants higher than 1 month of age were included in this study. Moreover, red cell degradation may be initiated in the fetus before delivery. The abrupt interruption in the net influx of bilirubin across the placenta from the fetus to the mother may also lead to further increase in the cord bilirubin [32]. The direct bilirubin RI was higher than values given in literature and manufacturer kit inserts (Table 8) but lower than values indicated for adult population. Similarly, the RI of total bilirubin was higher than values given for children and adults. But, it was significantly lower than values provided by manufacture kit inserts for newborns.

In this study, statistically significant variations were detected regarding albumin (*p = 0.0002*) values among newborns and infants (Figure 1g) even though the Harris and Boyd test failed to denote separated RIs. The slightly decreased concentration of albumin in newborns may not fully be explained by decreased synthesis as a result of liver immaturity since they are capable of endogenous albumin production even from early pregnancy on [33,34]. As a result, it could also be originated from other factors that govern albumin level like increased degradation, intravascular space and transcapillary escape [35,36]. The lower limit of albumin in newborns and infants was higher than that given in literature (Table 8) for these populations, but it was comparable to the adult values and kit inserts values except that it was lower than the values given for adult Ethiopian population. In contrast, the upper limit was higher than all the comparators used apart from the fact that it was lower than adult values determined in Ethiopia.

It has been indicated that a cord blood albumin value of >3.3 g/dl [37] and bilirubin value of <1.75 mg/dl [38] are presumed as safe levels in which there is minimal chance of neonatal jaundice. But, in this study, even the minimum value of albumin was higher than the aforementioned one. However, it needs further study to ascertain whether our newborns are really safe from subsequent occurrence of neonatal jaundice at the provided albumin level.

Maternal alcohol consumption during the periods of gestation was found to affect the values of AST (*p = 0.003*) and ALP (*p = 0.011*). The AST values were higher while ALP values were lower in newborns from mothers consuming alcohol during the period of gestation with separated RIs indicated for both variations (Table 7). These results need further investigation using increased sample sizes since the actual number of mothers consuming alcohol during the periods of gestation was actually 11 in this study.

In the infantile population, age dependent variation of ALP (*p = 0.002*) and GGT (*p = 0.000*) activity was detected in which the activities of both enzymes were higher in infants of age below 6 months though only the variation in GGT needed separated RI. The activity of GGT has been reported to decline in the postnatal periods reaching adult values by the age of 5–7 months [30]. Since majority of the infants below 6 months of age were on exclusive breast feeding, the increased GGT activity could partly be explained by absorption of maternal GGT mixed with breast milk [39].

Sex dependent variation of ALT was also revealed in which the activity of this enzyme is slightly higher in females with separated RIs indicated. Though separated RI is not indicated, ALP activity tended to be lower in infants on additional food to breast feeding. Albumin also showed age dependent variation in infants with separated RIs (Table 7). Infants of age group above 6 months were having higher values of albumin (*p = 0.001*) as a result of gradual increment through age [19].

## Conclusion

Even though all LFT analytes showed difference in newborns and infants, ALT and GGT activities only necessitated separated RIs. Some infantile and maternal factors like age, sex, feeding practice and maternal alcohol consumption during gestational periods were important factors that affected the values of analytes in serum of newborns and infants.

Almost all LFT analytes were different from previously reported values for other target population of similar age group, kit insert values and RIs given for adult populations. Hence, interpretation of LFT analytes in Ethiopian pediatric population sounds better to be performed by using such RIs taking the effect of infantile and maternal factors in to account.

## Competing interests

The authors declared no competing interests in this research. In fact, the research was financially supported by Addis Ababa University; and other non-financial supports were also obtained from Mesroy international plc (Reagents) and Medcalc Software Company (statistical software).

## Authors' contributions

MM, TT and SK have participated in the conception and design of the study. MY and TT have participated in the selection of study participants. MM, PN, SA and TG have participated in the laboratory analysis and acquisition of data. MM, TT, MY and SK have participated in preparing and critically reviewing the draft manuscript. All authors have read and approved the final manuscript.
